# Supplementary material for: An open-source framework for large-scale, flexible evaluation of biomedical text mining systems
Source: J Biomed Discov Collab. 2008 Jan 29;3:1. doi: 10.1186/1747-5333-3-1 (PMC2276192; doi:10.1186/1747-5333-3-1)
Supplement: Additional file 1 — Evaluation platform source code. The additional file contains the software evaluation framework discussed in this paper. The most current release can be downloaded from SourceForge.net [1]. See the accompanying README file for instructions on installation and use. Also included in the distribution is a collection of UIMA wrappers for some commonly used BioNLP tools and annotated corpora. [file 1747-5333-3-1-S1.zip › bionlp_uima_repository-1.0.1/docs/api/src-html/edu/uchsc/ccp/uima/ae/util/converter/ClassMentionConverter_AE.html]

```
001    package edu.uchsc.ccp.uima.ae.util.converter;
002    
003    import java.util.Arrays;
004    import java.util.HashSet;
005    import java.util.Iterator;
006    import java.util.Set;
007    
008    import org.apache.uima.analysis_engine.ResultSpecification;
009    import org.apache.uima.analysis_engine.annotator.AnnotatorConfigurationException;
010    import org.apache.uima.analysis_engine.annotator.AnnotatorContext;
011    import org.apache.uima.analysis_engine.annotator.AnnotatorContextException;
012    import org.apache.uima.analysis_engine.annotator.AnnotatorInitializationException;
013    import org.apache.uima.analysis_engine.annotator.AnnotatorProcessException;
014    import org.apache.uima.analysis_engine.annotator.JTextAnnotator_ImplBase;
015    import org.apache.uima.jcas.JCas;
016    
017    import edu.uchsc.ccp.uima.annotation.CCPDocumentInformation;
018    import edu.uchsc.ccp.uima.annotation.CCPTextAnnotation;
019    import edu.uchsc.ccp.uima.mention.CCPClassMention;
020    
021    /**
022     * Converts the class mentions of the user supplied types to the target mantion type
023     * 
024     * @author williamb
025     * 
026     */
027    public class ClassMentionConverter_AE extends JTextAnnotator_ImplBase {
028    
029        public static final String PARAM_TARGET_MENTION = "ToMentionType";
030    
031        public static final String PARAM_MENTION_TYPES_TO_CONVERT = "MentionTypesToConvert";
032        
033        private String targetMentionType = null;
034    
035        private Set<String> mentionTypesToConvert;
036        
037        @Override
038        public void initialize(AnnotatorContext ac) throws AnnotatorInitializationException, AnnotatorConfigurationException {
039            /* read in input parameters */
040            try {
041                targetMentionType = (String) ac.getConfigParameterValue(PARAM_TARGET_MENTION);
042                try {
043                mentionTypesToConvert = new HashSet<String>(Arrays.asList((String[]) ac.getConfigParameterValue(PARAM_MENTION_TYPES_TO_CONVERT)));
044                } catch (NullPointerException npe) {
045                    mentionTypesToConvert = new HashSet<String>();
046                }
047                    System.err.println("Initialized ClassMentionConverter: " + mentionTypesToConvert + " --> " + targetMentionType);
048            } catch (AnnotatorContextException e) {
049                e.printStackTrace();
050            }
051            super.initialize(ac);
052        }
053    
054        /**
055         * cycle through all annotations and change the mention if appropriate
056         */
057        public void process(JCas jcas, ResultSpecification arg1) throws AnnotatorProcessException {
058    
059            /* get document ID */
060            Iterator docInfoIter = jcas.getJFSIndexRepository().getAnnotationIndex(CCPDocumentInformation.type).iterator();
061            String documentID = "-1";
062            if (docInfoIter.hasNext()) {
063                CCPDocumentInformation srcDocInfo = (CCPDocumentInformation) docInfoIter.next();
064                documentID = srcDocInfo.getDocumentID();
065            }
066            
067            System.err.println("Converting classes for: " + documentID);
068            
069            Iterator annotIter = jcas.getJFSIndexRepository().getAnnotationIndex(CCPTextAnnotation.type).iterator();
070    
071            while (annotIter.hasNext()) {
072                Object possibleAnnot = annotIter.next();
073                if (possibleAnnot instanceof CCPTextAnnotation) {
074                    CCPTextAnnotation ccpTA = (CCPTextAnnotation) possibleAnnot;
075                    CCPClassMention ccpCM = ccpTA.getClassMention();
076                    String currentMentionType = ccpCM.getMentionName();
077                    if (mentionTypesToConvert.contains(currentMentionType)) {
078                        ccpCM.setMentionName(targetMentionType);
079                    }
080                } else {
081                    System.err.println("WARNING -- ClassMentionConverter: CCPTextAnnotation expected but instead got "
082                            + possibleAnnot.getClass().getName());
083                }
084            }
085    
086            
087            System.err.println("Done converting classes for: " + documentID);
088        }
089    
090    }
```
